# A draft genome of the neritid snail *Theodoxus fluviatilis*

**DOI:** 10.1093/g3journal/jkad282

**Published:** 2023-12-09

**Authors:** Laura Iris Regina Fuchs, Jan Knobloch, Amanda Alice Wiesenthal, Janina Fuss, Soeren Franzenburg, Montserrat Torres Oliva, Christian Müller, Christopher W Wheat, Jan-Peter Hildebrandt

**Affiliations:** Animal Physiology and Biochemistry, Zoological Institute and Museum, University of Greifswald, Felix Hausdorff-Strasse 1, D - 17489 Greifswald, Germany; Animal Physiology and Biochemistry, Zoological Institute and Museum, University of Greifswald, Felix Hausdorff-Strasse 1, D - 17489 Greifswald, Germany; Animal Physiology and Biochemistry, Zoological Institute and Museum, University of Greifswald, Felix Hausdorff-Strasse 1, D - 17489 Greifswald, Germany; Marine Biology, University of Rostock, Albert-Einstein-Straße 3, D - 18059 Rostock, Germany; Institute of Clinical Molecular Biology, Kiel University (CAU), University Hospital Schleswig Holstein, Rosalind-Franklin-Strasse 12, D - 24105 Kiel, Germany; Institute of Clinical Molecular Biology, Kiel University (CAU), University Hospital Schleswig Holstein, Rosalind-Franklin-Strasse 12, D - 24105 Kiel, Germany; Institute of Clinical Molecular Biology, Kiel University (CAU), University Hospital Schleswig Holstein, Rosalind-Franklin-Strasse 12, D - 24105 Kiel, Germany; Animal Physiology and Biochemistry, Zoological Institute and Museum, University of Greifswald, Felix Hausdorff-Strasse 1, D - 17489 Greifswald, Germany; Department of Zoology, Stockholm University, Svante Arrheniusväg 18 B, S-10691 Stockholm, Sweden; Animal Physiology and Biochemistry, Zoological Institute and Museum, University of Greifswald, Felix Hausdorff-Strasse 1, D - 17489 Greifswald, Germany

**Keywords:** genome, neritid snail, *Theodoxus fluviatilis*, osmotolerance, changes in environmental salinity, ecotypes

## Abstract

The neritid snail *Theodoxus fluviatilis* is found across habitats differing in salinity, from shallow waters along the coast of the Baltic Sea to lakes throughout Europe. Living close to the water surface makes this species vulnerable to changes in salinity in their natural habitat, and the lack of a free-swimming larval stage limits this species’ dispersal. Together, these factors have resulted in a patchy distribution of quite isolated populations differing in their salinity tolerances. In preparation for investigating the mechanisms underlying the physiological differences in osmoregulation between populations that cannot be explained solely by phenotypic plasticity, we present here an annotated draft genome assembly for *T. fluviatilis*, generated using PacBio long reads, Illumina short reads, and transcriptomic data. While the total assembly size (1045 kb) is similar to those of related species, it remains highly fragmented (*N* scaffolds = 35,695; N50 = 74 kb) though moderately high in complete gene content (BUSCO single copy complete: 74.3%, duplicate: 2.6%, fragmented: 10.6%, missing: 12.5% using metazoa *n* = 954). Nevertheless, we were able to generate gene annotations of 21,220 protein-coding genes (BUSCO single copy complete: 65.1%, duplicate: 16.7%, fragmented: 9.1%, missing: 9.1% using metazoa *n* = 954). Not only will this genome facilitate comparative evolutionary studies across Gastropoda, as this is the first genome assembly for the basal snail family Neritidae, it will also greatly facilitate the study of salinity tolerance in this species. Additionally, we discuss the challenges of working with a species where high molecular weight DNA isolation is very difficult.

## Introduction

Sea level rise due to the current climate change triggers salination of coastal water bodies. This effect may be aggravated by evaporation of water from shallow coastal ponds during dry periods. On the other hand, intense rainfall may very quickly result in strong dilution effects. Phenotypic plasticity/elasticity remains a key feature in immobile or slow-moving organisms to survive such alterations in their ever-changing environments (Hildebrandt *et al.* 2018). The underlying mechanisms may involve environmentally driven alterations in gene expression, protein modifications, e.g. post-translational modifications, leading to altered enzyme activities, or re-direction of metabolic processes (Lockwood and Somero 2011; Woods 2014).

Here we focus upon the neritid snail *Theodoxus fluviatilis* (Linneaus, 1758), which exemplifies moderately stationary organisms in these habitats. *T. fluviatilis* forms fairly isolated populations because reproduction of this species does not involve free-swimming larval stages. Rather, the juvenile snail will hatch directly from its egg capsule that has been attached to hard substrate by the mother. Accordingly, dispersal of this species is rather limited (Bondesen 1940; Kirkegaard 2006). However, the number of individuals in each population may be quite high, with an estimated average of 1,160 individuals per square meter for Lake Esrom, Denmark (Kirkegaard 2006).

*T. fluviatilis* has formed regional subgroups in northern Germany that differ somewhat in their shell patterns and sizes (Kangas and Skoog 1978; Zettler *et al.* 2004; Wiesenthal *et al.* 2018). The freshwater (FW) ecotype inhabits lakes, ponds, and streams while the brackish water (BW) ecotype lives along the shore of the Baltic Sea. Despite sharing the same phylogenetic lineage (based on cytochrome c oxidase subunit I and 16S rRNA), (Bunje 2005; Bunje and Lindberg 2007), these ecotypes exhibit clear differences in osmotolerance (Symanowski and Hildebrandt 2010; Wiesenthal *et al.* 2018) and in the mode of accumulating organic osmolytes under hyperosmotic stress (Wiesenthal *et al.* 2019). These differences appear to be genetic in origin, since the osmotolerance ranges of the 2 ecotypes have never been observed to converge despite prolonged salinity acclimations (Wiesenthal *et al.* 2018).

Apart from mitochondrial genomes of other neritid snail species (Fukumori *et al.* 2016; Fukumori *et al.* 2020; Feng *et al.* 2021; Miao *et al.* 2022), there is no genomic information available for any species with close phylogenetic relationship to *Theodoxus*. Despite a number of snail genomes having been published to date, neither the family Neritidae nor the clade Neritimorpha are represented. The currently published snail genomes are all roughly 400 million years divergent from *T. fluviatilis* (Table 1) (Jin *et al*. 2022; Pardos-Blas *et al.* 2021; Gregory 2003; Kumar *et al.* 2017). Currently nothing is known about the genetic basis of local adaptation in the FW and BW ecotypes. Gaining such insights has the potential to tell us more about local adaptations and maybe even early speciation in general. Although *T. fluviatilis* has also been recognized as a species of interest by the Darwin Tree of Life, its genome has proven to be very difficult to assemble. This is consistent with challenges inherent in working with mollusk genomes, such as high concentrations of polysaccharides and highly variable genome sizes between related groups (Davison and Neiman 2021; Gomes-dos-Santos *et al*. 2020). In order to move this research forward, we report a draft genome for *T. fluviatilis*. Further, we describe our pipeline for navigating the challenges involved in assembling and annotating the genome of this species in detail—such as residual mucus in DNA extracts—as a roadmap for other snail genomics projects.

**Table 1.** Table showing the amount of time each snail species is divergent from *T. fluviatilis* according to www.Timetree.org.

*Theodoxus fluviatilis* vs species	GenBank accession nr.	Bioproject nr.	Median time (MYA)	Adjusted time (MYA)
*Aplysia californica*	GCA_000002075.2		447	476
*Bellamya* sp.		PRJNA818874	391	434
*Conus ventricosus*		PRJNA678883	447	476
*Littorina saxatilis*		PRJNA850123	447	476
*Haliotis rufescens*	GCA_023055435.1		422	476
*Biomphalaria glabrata*	GCA_000457365.2		391	434

## Materials and methods

### Experimental animals

Adult snails were collected at 3 freshwater and 3 brackish water sites in northern Germany (c.f. Wiesenthal *et al.* 2018) with the exception that the animals of the Ludwigsburg/Loissin population were replaced by those from the island of Riems (54°11′00.1″N 13°21′10.2″E). Snails were kept in large tanks at room temperature (20–22°C) under a 12 h:12 h light:dark cycle with constant aeration. Pebbles and substrate from the original locations were added to the tanks to ensure proper growth of diatoms, the major nutrient source for these snails. Natural salinity conditions were maintained by replacing small portions of the original media with saline prepared by dissolving “Tropic Marine Classic” sea salt (Dr. Biener GmbH, Wartenberg, Germany) in deionized water. Osmolality was regularly measured using a Vapro 5520 osmometer (Wescor Inc., Logan, UT, USA).

Some of the animals whose RNA would be later isolated for transcriptomic analyses were subjected to transfer experiments starting 1 week after collection. For the duration of the transfer experiments, the snails were kept in small glass aquaria with 1 l of water (up to 12 individuals each) and some pebbles. Animals were acclimated to increasing (FW as well as BW animals) or decreasing (BW animals) external salinities in a stepwise manner as described previously (Wiesenthal *et al.* 2018).

### RNA extraction, cDNA library construction, and Mi-Seq sequencing

The whole procedure of RNA extraction, cDNA library construction, and Mi-seq sequencing was already described in great detail in Knobloch *et al.* (2023). Briefly, a pool of foot muscle preparations of 24 individuals that were collected at freshwater and brackish water locations in Northern Germany and exposed to different salinities under laboratory conditions were used to extract total RNA. Sequencing and assembling were carried out by GATC Biotech AG (Konstanz, Germany) using an Illumina MiSeq2500 system with 300 bp paired-end reads. The sequence reads were adapter trimmed and quality filtered using BBDuk version 34.86 (Bushnell 2015). Both raw data (AccNo. SRR15300633) and assembled contigs (AccNo. GKDH00000000.1) were submitted to GenBank.

### Isolation of DNA from foot muscle tissue and library preparation for sequencing

#### PacBio whole genome sequencing

DNA extraction, sample and library preparations, as well as PacBio whole genome sequencing were carried out in the Competence Centre for Genomic Analysis (Kiel, Germany). Tissue of the foot muscle prepared from 1 BW individual was disrupted in liquid nitrogen using a mortar and pestle, followed by an additional proteinase K digestion. The tissue samples were frozen in liquid nitrogen in hope to break up the mucus secreted by the snails’ foot muscle tissue. As per discussions with colleagues prior to DNA sequencing, it was decided to use PacBio, instead of Nanopore, because even with the disruption of the tissue samples in liquid nitrogen the residual mucus left in the DNA extract samples would cause clogging of the pores causing sequencing runs to fail or produce unreliable results. The DNA extraction was performed using the MagAttract HMW DNA Kit (Qiagen) according to the manufacturer's protocol. The DNA was stored at −80°C until use. A PacBio SMRTbell express template prep kit 2.0 was prepared with the modification for low input samples without size selection. The library was sequenced on 4 SMRTcells (SMRTCell 1M v3), sequencing chemistry 2.1.

#### Illumina whole genome sequencing

For Illumina whole genome sequencing, 24 male and 26 female adult snails from 6 different locations were selected (Supplementary Table 1), the foot muscles individually dissected and placed in 2 ml microcentrifuge tubes. The dissected tissue was immediately frozen in liquid nitrogen and stored at −80°C until further processing. Prior to DNA extraction, 180 µl of the lysis Buffer ATL (DNeasy Blood and Tissue Kit, Qiagen, Valenicia, CA) was added to each sample and the tissue was homogenized (T8-ULTRA-TURRAX, IKA-Werke, Staufen, Germany). DNA extraction was performed according to the manufacturer's protocol. The samples were sent to the Competence Centre for Genomic Analysis (Kiel, Germany) where the DNA was quality-controlled on Tape Station Genomic Tape and quantified via fluorometric measurement (Qubit). Library preparation and sequencing were carried out in the Competence Centre for Genomic Analysis (Kiel, Germany). Library preparation was done using the Illumina DNA Prep Kit according to the manufacturer's protocol (i.e. transposase integration). Sequencing of the libraries was performed on 2 lanes of an Illumina NovaSeq 6000 S4 machine, using 2 × 150 bp reads on a v1.5 Flowcell.

#### De novo genome assembly and assembly improvement

The raw PacBio reads were de novo assembled using Flye v2.8 (Kolmogorov *et al.* 2019) in the *PacBio-raw* mode and assessed using Benchmarking Universal Single-Copy Orthologs (BUSCO) v5.3.2 (Simão *et al.* 2015; Manni *et al.* 2021). Using the metazoan database as a reference yielded better results than using the mollusk database which may be explained by the fact that the mollusk database only has a few entries and the neritid snails are evolutionarily distant from the mollusk genomes contained in this database (Cunha and Giribet 2019). It was decided to move forward with the Flye-Assembly after several other assembly algorithms had been tried and failed to produce satisfying results (Supplementary Table 2). The initial genome assembly was screened for contamination using BlobTools blobtoolkit v3.3.4 (Laetsch and Blaxter 2017). No major contamination was detected (Supplementary Fig. 1). After a first round of polishing using Racon v1.4.3 (Vaser *et al.* 2017) utilizing all 50 (from 24 male and 26 female snails) low-coverage Illumina short reads, the RNAseq data were mapped to the polished assembly utilizing HISAT 2.2.1 (Kim *et al.* 2019) and the RNAseq reads were used for scaffolding via P_RNA_scaffolder with default settings (Zhu *et al.* 2018). The scaffolded genome was then polished 2 consecutive times with Pilon v1.24 (Walker *et al.* 2014), using the mapping of Illumina short reads. This final genome assembly was again checked for contamination using BlobTools blobtoolkit v.3.3.4 (Laetsch and Blaxter 2017) and no major contaminations were detected (Supplementary Fig. 2). We would like to remind the reader at this point that one of the caveats in working with *T. fluviatilis* is, that the species is highly divergent and no genomic or transcriptomic information can so far be found on close relatives of this species. Repeats were identified and masked using RepeatMasker version 4.1.4 (A.F.A. Smit, R. Hubley & P. Green RepeatMasker at http://repeatmasker.org). Annotation was performed utilizing Braker3 (Lomsadze *et al.* 2005; Stanke *et al.* 2006; Gotoh 2008; Stanke *et al.* 2008; Iwata and Gotoh 2012; Lomsadze *et al.* 2014; Buchfink *et al.* 2015; Hoff *et al.* 2016; Hoff *et al.* 2019; Kovaka *et al.* 2019; Bruna *et al.* 2020; Pertea and Pertea 2020; Bruna *et al.* 2021) with a custom molluskan protein dataset and the RNAseq data. The RNAseq data was mapped to the softmasked genome using HISAT2 (Kim *et al.* 2019), while the custom molluskan protein dataset consisted of protein data downloaded from NCBI for 6 mollusk species (Table 2). Additionally, we used TSEBRA (Gabriel *et al.* 2021) post hoc to enforce the gene set created by Augustus (Stanke *et al.* 2004; Stanke and Morgenstern 2005; Stanke *et al.* 2006) and filter out single exon genes. Lastly we utilized AGAT script agat_sp_keep_longest_isoform.pl v0.6.0 (Dainat 2022; https://github.com/NBISweden/AGAT) to only keep the longest isoforms. Finally, a functional annotation of the annotated proteins was performed using EggNOG-mapper version 2.0.1. (Huerta-Cepas *et al.* 2019; Cantalapiedra *et al.* 2021).

**Table 2.** Mollusk species used to generate the protein database for genome annotation.

Species	Number of proteins	Refseq accession number	GenBank accession number	Technology	N50	BUSCOs [%] (complete and fragmented)
*Aplysia californica*	26,656	GCF_000002075.1	GCA_000002075.2	Illumina Hi-Seq	917,541	
*Crassostrea gigas*	46,748	GCF_000297895.1	GCA_000297895.2	Illumina Hi-Seq	286,862	88
*Biomphalaria glabrata*	36,675	GCF_000457365.1	GCA_000457365.2	454 FLX Titanium	48,076	88.4
*Octopus bimaculoides*	23,994	GCF_001194135.1	GCA_001194135.2	Illumina Hi-Seq	96,881,196	94.6
*Crassostrea virginica*	60,213	GCF_002022765.2	GCA_002022765.4	PacBio_RSII	75,944,018	98.2
*Mizuhopecten yessoensis*	41,567	GCF_002113885.1	GCA_002113885.2	Illumina Hi-Seq	803,631	97.1

BUSCOs *N*_Metazoa_ = 954.

## Results and discussion

### Assembly

**Fig. 1.**
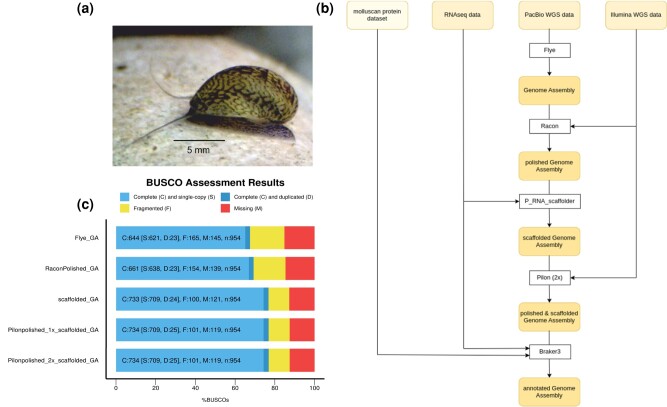
a) A picture of *Theodoxus fluviatilis* taken by Amanda A. Wiesenthal. b) Data and pipeline used to create the genome assembly of *T. fluviatilis*. c) BUSCO results after each step in the pipeline (b).

In total, 14 Gb of PacBio reads with a raw read length of about 5.7 Kb were generated on 4 SMRT cells. The initial assembled genome comprised 1,045,476,465 bp, spread across 40,380 contigs with an N50 of 55,460 bp. The Darwin Tree of Life has estimated the genome size of *T. fluviatilis* via 3 different methods, which vastly differ in their results. However the hic-arima2 results in a haploid genome size of 1,288,516,633 bp (https://tolqc.cog.sanger.ac.uk/darwin/molluscs/Theodoxus_fluviatilis), which is quite close to our assembly size with 13× coverage. A BUSCO assessment revealed only moderate genetic content [Completeness: 67.5% (single copy complete: 65.1%, duplicated: 2.4%), fragmented: 17.3%, missing: 15.2%], with completeness similarly checked after each step. After polishing the assembly using Racon, the contig N50 was 55,347 bp with BUSCO scores slightly improved [BUSCO C: 69.3% (S: 66.9%, D: 2.4%), F: 16.1%, M: 14.6%] (Fig. 1). Using RNA reads to scaffold the polished genome assembly further increased contiguity (N50 = 73,835 bp) and content [BUSCO C: 76.8% (S: 74.3%, 2.6%), F: 10.6%, M: 12.5%]. Additional polishing runs with Pilon improved the assembly only slightly, resulting in our final de novo assembly of 35,695 contigs, with an N50 = 73,828 bp and moderate BUSCO scores [BUSCO C: 76.9% (S: 74.3%, D: 2.6%), F: 10.6%, M: 12.5%].

### Annotation

HISAT2 aligned 73.13% of the RNAseq reads to the genome. In combination with the RNAseq reads, protein datasets from 6 different molluskan species (Table 2)—comprising a total of 235,853 proteins—were used for the annotation (Fig. 1). RepeatMasker identified and masked a total of 510,160,421 bp repeat sequences, which corresponds to 48.92% of the *T. fluviatilis* genome. The most common types of transposable and repetitive elements were long interspersed nuclear elements (LINEs), which make up 7.74% of the genome. Using the softmasked genome, annotation using Braker3 and TSEBRA resulted in 71,293 predicted proteins. This suspected overprediction was reduced by employing the AGAT script agat_sp_keep_longest_isoform.pl, resulting in 64,459 predicted proteins. The quality of the gene annotation was assessed by BUSCO [C: 81.8% (S: 65.1%, D: 16.7%), F: 9.1%, M: 9.1%]. EggNOG-mapper identified 21,220 functional protein-coding genes.

Compared to other gastropod species, e.g. *Aplysia californica* (Cooper, 1863) with 26,656 proteins or *Biomphalaria glabrata* (Say, 1818) with 36,675 proteins, *T. fluviatilis* has a large number of protein-coding genes. While this discrepancy might be explained by the great phylogenetic distance between these gastropod species, another factor to consider is the type of annotation algorithm that was used for the different species. *A. californica* and *B. glabrata* genomes were annotated using the NCBI Eukaryotic Genome Annotation Pipeline. This pipeline uses an HMM-based gene prediction program called Gnomon, which—in comparison with Braker3—tends to underpredict protein-coding gene content (Hoff *et al.* 2019). On the other hand for the pacific oyster *Crassostrea gigas* 60,213 proteins (Table 2) are reported, which is comparable to the number of predicted proteins for *T. fluviatilis* with 64,459. Another challenge in working with gastropod genomes is the high repeat content, as reported for *Radix auricularia* (70%) (Schell *et al.* 2017). However, this was not quite the case for our *T. fluviatilis* genome, which shows a total interspersed repeat content of 38.09% (Table 3). Regarding the caveats DNA extractions pose in molluskan species, we are confident that by shock-freezing the samples prior to DNA extraction the polysaccharides were broken up sufficiently to not hinder either PacBio or Illumina sequencing technologies. However, we have since started to use the E.Z.N.A Mollusc DNA Kit (Omega Bio-tek, Inc., Norcross, GA) instead of the Illumina DNeasy Blood and Tissue Kit (Qiagen, Valenicia, CA) which is specifically designed for use on tissue samples rich in mucopolysaccharides. Still the genomic data used in this assembly was of low coverage, which might certainly play a role in the low BUSCO gene counts.

**Table 3.** Table of assembly and annotation summary statistics.

Genome assembly and annotation summary	
Genome assembly statistics	
Total length (bp)	1,042,808,253
Contig N50 length (bp)	73,828
Longest contig length (bp)	725,830
Number of contigs	35,695
Percentage of non-ATGC characters	0.040
Repetitive sequences (%)	
LINE (bp)	80,752,569 (7.74%)
SINE (bp)	403,990 (0.04%)
LTR (bp)	17,794,117 (1.71%)
Simple repeat (bp)	106,312,912 (10.19%)
Unclassified (bp)	268,962,851 (25.79%)
Total interspersed repeats (bp)	397,175,243 (38.09%)

## Conclusion

Here we report a first draft genome of the neritid snail *T. fluviatilis.* We are confident that we achieved the best genome assembly possible with the use of all the data generated to date. Although this draft genome is fragmented, it will nevertheless work well as a reference for mapping individual re-sequencing data and allow us to detect population-specific genetic differences, especially those whose gene products affect the ability of the animals to adjust to salinity changes in their environments.

## Data Availability

The assembled genome has been deposited at DDBJ/ENA/GenBank under the accession JAWQFJ000000000. Genomic raw reads (Illumina and PacBio) are available at NCBI SRA under the accession PRJNA997091. Both RNAseq raw data (AccNo. SRR15300633) and assembled contigs (AccNo. GKDH00000000.1) were submitted to GenBank. Scripts and supplementary files, as well as the assembled genome and annotation, are also available at figshare (https://doi.org/10.6084/m9.figshare.23623014.v8).
